# NIR-chemometric approaches for evaluating carbonization characteristics of hydrothermally carbonized lignin

**DOI:** 10.1038/s41598-021-96461-x

**Published:** 2021-08-20

**Authors:** Sung-Wook Hwang, Un Taek Hwang, Kyeyoung Jo, Taekyeong Lee, Jinseok Park, Jong-Chan Kim, Hyo Won Kwak, In-Gyu Choi, Hwanmyeong Yeo

**Affiliations:** 1 Research Institute of Agriculture and Life Sciences, Seoul National University, 1 Gwanak-ro, Gwanak-gu, Seoul, 08826 Republic of Korea; 2 Department of Forest Sciences, College of Agriculture and Life Sciences, Seoul National University, 1 Gwanak-ro, Gwanak-gu, Seoul, 08826 Republic of Korea; 3 Department of Agriculture, Forestry and Bioresources, College of Agriculture and Life Sciences, Seoul National University, 1 Gwanak-ro, Gwanak-gu, Seoul, 08826 Republic of Korea

**Keywords:** Energy science and technology, Materials science

## Abstract

The aim of this study is to establish prediction models for the non-destructive evaluation of the carbonization characteristics of lignin-derived hydrochars as a carbon material in real time. Hydrochars are produced via the hydrothermal carbonization of kraft lignins for 1–5 h in the temperature range of 175–250 °C, and as the reaction severity of hydrothermal carbonization increases, the hydrochar is converted to a more carbon-intensive structure. Principal component analysis using near-infrared spectra suggests that the spectral regions at 2132 and 2267 nm assigned to lignins and 1449 nm assigned to phenolic groups of lignins are informative bands that indicate the carbonization degree. Partial least squares regression models trained with near-infrared spectra accurately predicts the carbon content, oxygen/carbon, and hydrogen/carbon ratios with high coefficients of determination and low root mean square errors. The established models demonstrate better prediction than ordinary least squares regression models.

## Introduction

Lignin is an abundant, naturally occurring organic material. Increasing environmental restrictions and fossil fuel prices have rendered lignocellulosic biomass an important source of renewable materials. Most of the currently commercialized carbon materials are coal based, and carbon dioxide and toxic gases generated during mining and refining can result in climate change, in addition to environmental pollution. By contrast, lignin is an eco-friendly and economical raw material that is mass produced as a byproduct of pulping in the paper industry. Lignins produced in industry are used primarily as a fuel for generating electricity and heat^1^. However, recent studies have been conducted to produce various high-value-added products from lignins, such as carbon fibers, activated carbon, vanillin, phenol derivatives, and phenolic resins^2–5^.

Hydrothermal carbonization (HTC), one of the major approaches to utilizing lignin, is a thermochemical conversion process from biomass for producing coal-like products^6^. Compared with pyrolysis, which is another important category of biomass conversion, HTC offers the advantage of relatively low processing requirements, stability, and nontoxicity owing to its low process temperature (180–250 °C). Hydrochars produced by the HTC of lignin can be used in various applications, including lightweight polymer composites, absorbents, electrochemical devices, energy devices, batteries, and automotive^1,7–9^, not for low-cost fuels.

Although the carbonization characteristics of lignin-derived hydrochars must be evaluated, real-time measurements are not possible through conventional elemental analysis based on high-temperature combustion and gas chromatography. Owing to its advantages of fast and non-destructive measurements, simple sample preparation, and low cost^10,11^, near-infrared spectroscopy (NIRS) may be a promising alternative for overcoming the limitations of conventional methods. In combination with multivariate analysis, NIRS is suitable for physical and chemical analyses of wood^12–15^, as well as for wood identification^16,17^.

The aim of this study is to establish accurate models for predicting the carbonization characteristics of kraft-lignin-derived hydrochars. The hydrochars were produced under various HTC conditions, and their elemental compositions were determined via elemental analysis. Principal component analysis (PCA) and partial least squares regression (PLSR) were employed to analyze the carbonization characteristics from near infrared (NIR) spectra obtained from the hydrochars and to establish prediction models, respectively.

## Results and discussion

### Elemental analysis

The elemental compositions of the hydrochars produced by HTC are listed in Table 1. The carbon content (C wt%) of the control (uncarbonized) sample was 62.83 wt%, and this value increased with the HTC temperature and residence time. The HTC process at 175 °C for 1 h increased the carbon content by approximately 2.5 wt% compared with the control, and the process at 250 °C for 5 h increased by approximately 6.5 wt%. As the HTC temperature and residence time increased, i.e., as the reaction severity increased, more carbon-intensive hydrochars were produced.

**Table 1:** Elemental composition of kraft-lignin-derived hydrochars.

Sample		C (wt%)	H (wt%)	N (wt%)	S (wt%)	O^a^ (wt%)
Temp. (°C)	Time (h)					
Control		62.83	5.79	0.39	1.74	29.25
175	1	65.36	5.66	0.46	1.29	27.23
	2	65.76	5.65	0.40	1.24	26.95
	3	65.73	5.64	0.41	1.20	27.02
	5	65.82	5.63	0.41	1.21	26.93
200	1	65.73	5.52	0.41	1.28	27.06
	2	66.19	5.46	0.43	1.41	26.51
	3	66.25	5.55	0.42	1.24	26.53
	5	67.02	5.58	0.43	1.23	25.73
225	1	67.24	5.58	0.43	1.13	25.62
	2	67.43	5.58	0.42	1.13	25.44
	3	67.44	5.48	0.41	1.11	25.56
	5	68.11	5.51	0.43	1.12	24.82
250	1	68.23	5.48	0.45	1.12	24.73
	2	68.64	5.54	0.47	0.92	24.43
	3	69.12	5.47	0.49	0.92	24.00
	5	69.37	5.38	0.49	0.87	23.89

^a^O (wt%) = 100 − (C + H + N + S) (wt%).

**Figure 1:**
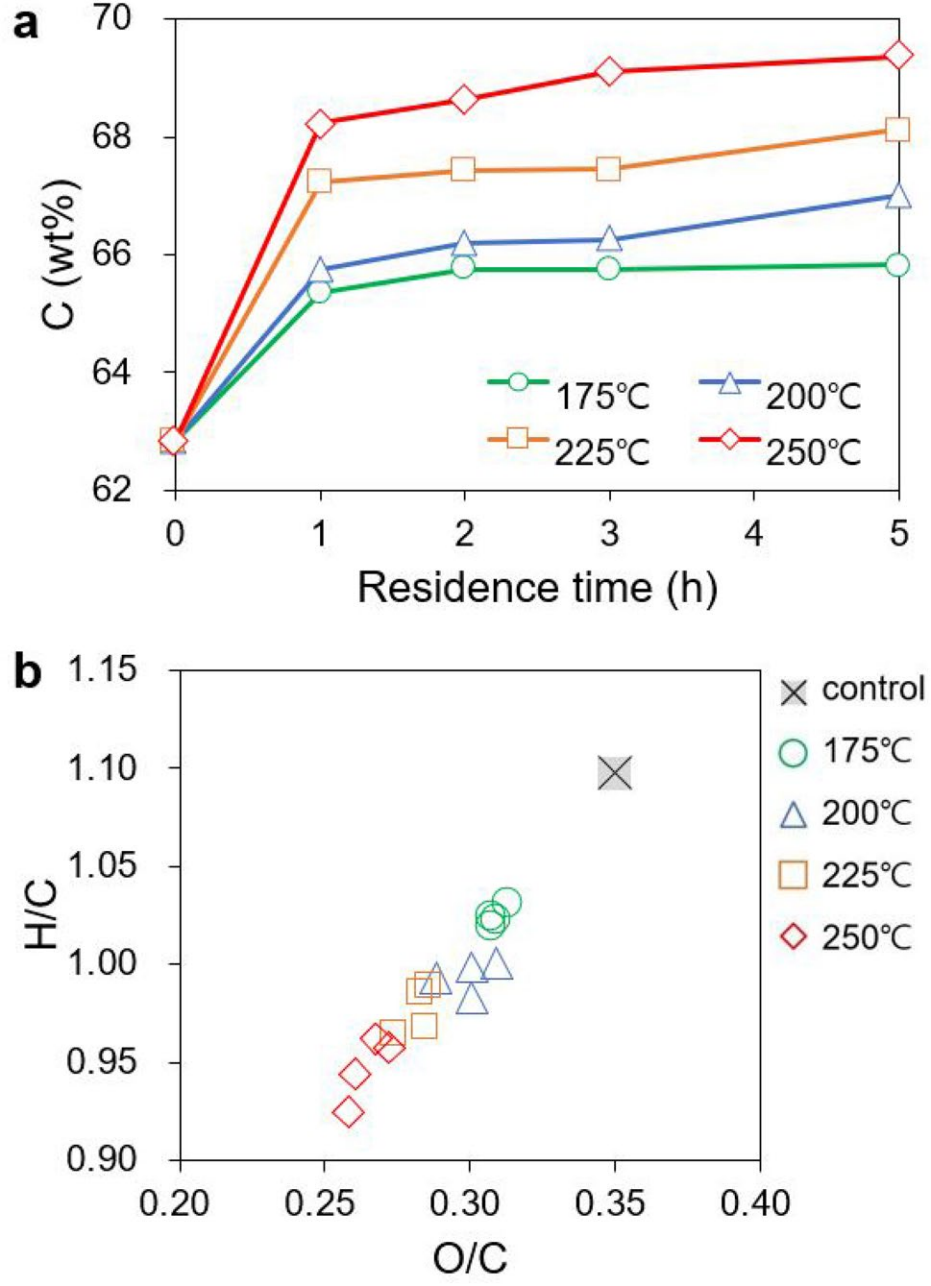
Carbonization characteristics of lignin-derived hydrochars. (**a**) Changes in carbon content of lignin samples with HTC temperature and residence time, and (**b**) its van Krevelen diagram, a plot of atomic O/C vs. atomic H/C.

As shown in Fig. 1a, the carbon content increased logarithmically with the residence time at each temperature. This suggests that temperature imposes a more prominent effect on the carbon content than the residence time in the HTC of lignin. The van Krevelen diagram (Fig. 1b), which shows the changes in the oxygen/carbon (O/C) and hydrogen/carbon (H/C) ratios, shows the formation of a carbon-intensive structure via HTC. As the reaction severity increased, both O/C and H/C decreased. This is because HTC is an exothermic process that reduces both the oxygen and hydrogen contents of the feed via dehydration and oxidation^6,18,19^.

### Spectral characteristics of HTC lignin

**Figure 2:**
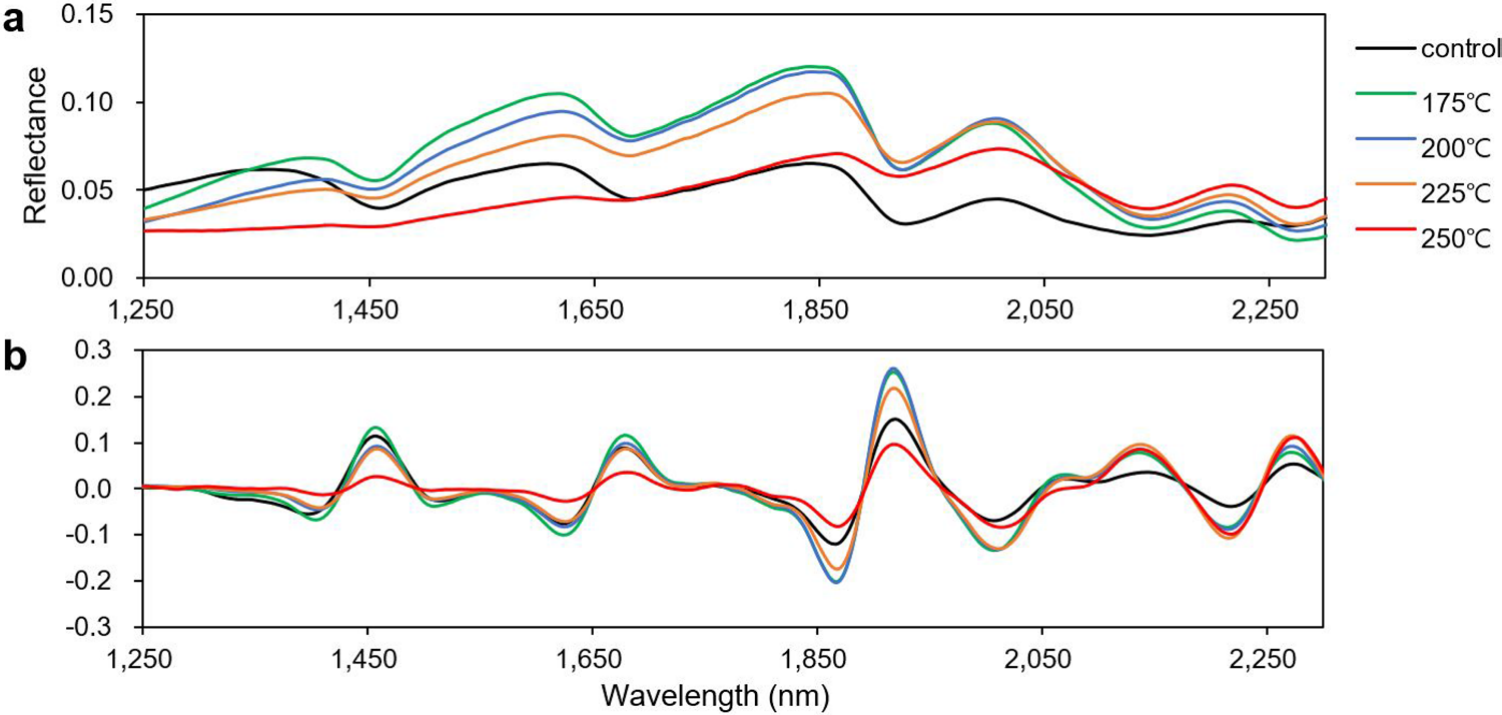
Original NIR spectra (**a**) and second-derivative spectra (**b**) with 1250–2300 nm region for hydrothermally carbonized kraft lignin and control samples.

Figure 2 shows the original NIR spectra and the second-derivative spectra of hydrochars in the spectral region of 1250–2300 nm. In the original spectra (Fig. 2a), it was difficult to distinguish the difference among the spectra by the HTC temperature, except that some peaks disappeared at 250 °C. By contrast, in the second-derivative spectra (Fig. 2b), not only was the baseline of the spectrum adjusted, but the peaks were amplified, rendering the difference among the spectra with HTC temperatures more evident. Some differences were observed at wavelengths of 1449, 1685, 1927, and 2132 nm. These bands, except for those at 1927 nm assigned to water^20^, were associated with lignin. The bands at 1449, 1685, and 2132 nm were assigned to the phenolic group, aromatic ring associated, and lignin, respectively^21–24^.

### PCA

**Figure 3:**
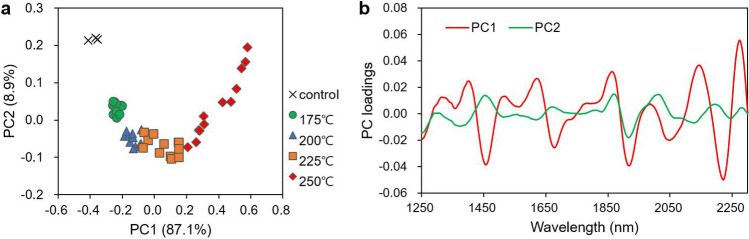
PCA score plot on first two PCs in second-derivative NIR spectra (**a**) and loadings of PCs (**b**). In score plot (**a**), percentages in parentheses of x- and y-axis titles are those of explained variables of corresponding PC.

Figure 3 shows a two-dimensional scatter plot of scores and loadings for the first two principal components (PCs) from the PCA of the second-derivative NIR spectra. The first two PCs constituted 96% of the total variance (Fig. 3a). In the plot, the scores for each HTC temperature were segmented into clusters. Because the scores for each HTC temperature were primarily aligned along the PC1 axis, it was discovered that this component contained information regarding the reaction severity, whereas PC2 and PC1 contained information that can determine whether HTC occurred in lignin.

In the PC loading plot (Fig. 3b), the two PCs indicated high loading values in the bands related to lignin, and among them, PC1 showed the highest values at 2132 and 2267 nm, both of which were assigned to lignin^23,24^. As shown from the second-derivative NIR spectra in Fig. 2b, the hydrochars and control indicated a clear spectral difference at 2132 and 2267 nm, where a higher HTC temperature resulted in a higher peak. PC2 indicated a relatively high loading at 1449 nm, which was assigned to the phenolic groups of lignin. It appeared that as the HTC temperature increased, the number of phenolic groups decreased, thereby increasing the carbon content. A decrease in the aromatic structure as the reaction severity increases has been reported in natural and HTC coals^25^.

### Regression models

**Figure 4:**
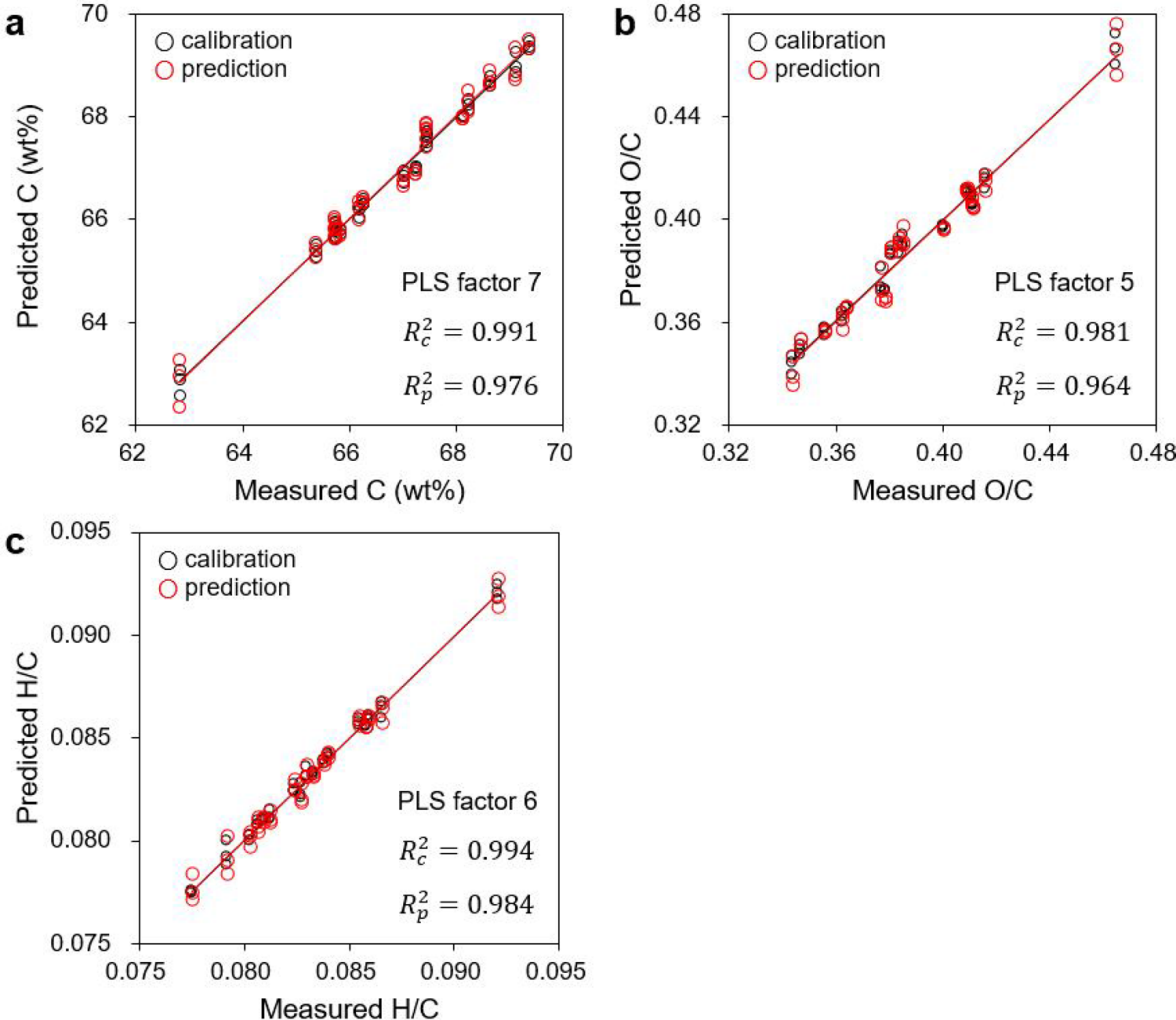
Scatter plots of prediction results for carbon content (**a**), O/C (**b**), and H/C (**c**) by PLSR models trained with second-derivative NIR spectra. *R*^2^_c_ coefficient of determination for calibration, *R*^2^_p_ coefficient of determination for prediction.

Regression models were established to predict the carbonization characteristics of kraft-lignin-derived hydrochars produced via HTC. Figure 4 shows the results of the PLSR models trained with the second-derivative NIR spectra for C (wt%), O/C, and H/C predictions. The models predicted C (wt%), O/C, and H/C with high coefficients of determination (*R*^2^) of 0.976, 0.964, and 0.984, respectively, demonstrating that PLSR with NIRS is promising for predicting the carbonization characteristics of hydrochars.

**Figure 5:**
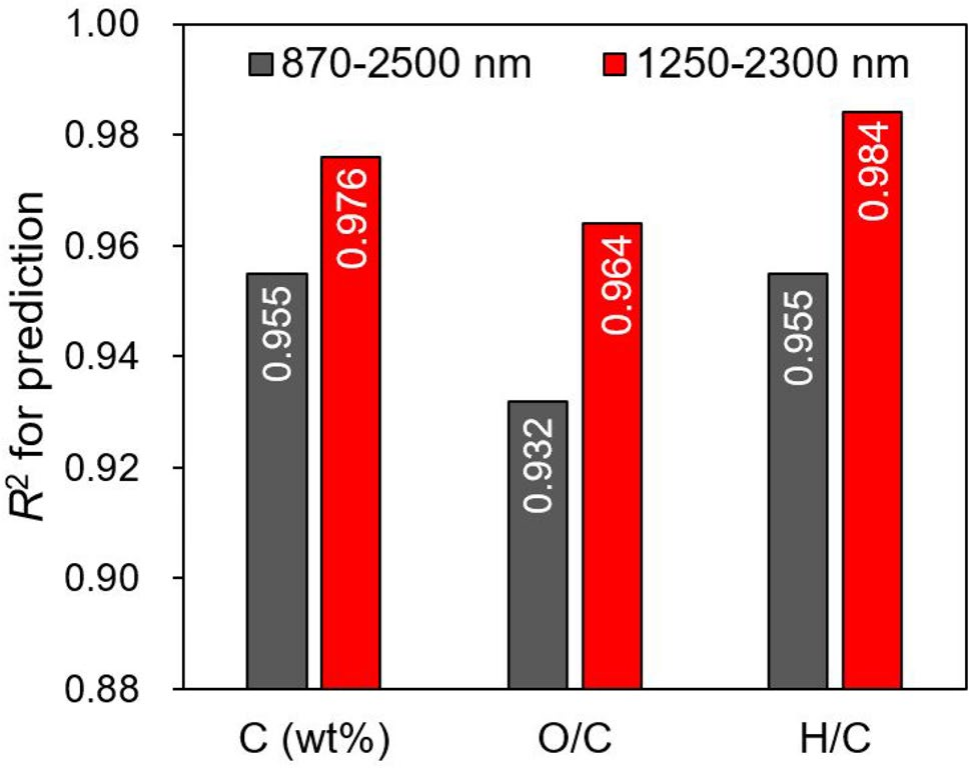
Comparison of coefficients of determination for prediction of PLSR models established in NIR spectral region of 870–2500 nm (full range) and 1250–2300 nm (selected in this study).

In this study, the spectral region of 1250–2300 nm was selected from the original range of 870–2500 nm and used to analyze the carbonization characteristics and establish prediction models. Although the unselected regions were noisy, some informative spectral bands related to lignin were present within these regions. Therefore, to determine whether the selection of the spectral region was appropriate, the prediction performance of the PLSR models in the second-derivative spectra with a full range of 870–2500 nm was investigated. As shown in Fig. 5, the selected spectral region (1250–2300 nm) yielded higher *R*^2^ values than those of the full spectral region (870–2500 nm) for all carbonization characteristics, thereby justifying the spectral selection.

Spectrum pretreatment contributed insignificantly to the improvement in the prediction performance of the PLSR models. Models trained with the second-derivative NIR spectra produced higher *R*^2^ values and lower root mean square errors (RMSEs) as well as reduced numbers of optimal partial least squares (PLS) factors than those trained with the original spectra (Table 2).

**Table 2:** Prediction performance for carbonization characteristics of PLSR models in NIR spectra with 1250–2300 nm region and comparison with OLSR models.

Output variable	Spectrum pretreatment	PLSR					OLSR			
		PLS factors	Calibration		Prediction		Calibration		Prediction	
			*R*^2^_c_	RMSEC	*R*^2^_p_	RMSEP	*R*^2^_c_	RMSEC	*R*^2^_p_	RMSEP
C (wt%)	Original	8	0.987	0.1840	0.973	0.2590	0.999	0.0003	0.953	0.3440
	2nd derivative	7	0.991	0.1486	0.976	0.2461	0.999	0.0003	0.940	0.3885
O/C	Original	6	0.974	0.0048	0.963	0.0057	0.999	0.0003	0.907	0.0091
	2nd derivative	5	0.981	0.0041	0.964	0.0056	0.999	0.0003	0.874	0.0106
H/C	Original	7	0.990	0.0003	0.984	0.0004	0.992	0.0003	0.966	0.0006
	2nd derivative	6	0.994	0.0003	0.984	0.0004	0.992	0.0003	0.912	0.0010

*PLSR* partial least squares regression, *OLSR* ordinary least squares regression, *R*^*2*^_*c*_ coefficient of determination for calibration, *R*^*2*^_*p*_ coefficient of determination for prediction, *RMSEC* root mean square error of calibration, *RMSEP* root mean square error of prediction.

To evaluate the prediction performance of the PLSR models, ordinary least squares regression (OLSR) models trained with identical spectral data were developed, and their performance comparisons are listed in Table 2. The PLSR models outperformed the OLSR models in predicting all the carbonization characteristics tested. In contrast to OLSR, which used all of the selected NIR spectral regions as input variables (165-dimensional vector), PLSR yielded better performances, even from variables (PLS factors) scaled down to eight or fewer. We attribute this result to the presence of non-informative or non-contributing spectral bands for the prediction in the NIR spectra. In contrast to spectral bands assigned to the phenolic group (1449 nm), aromatic ring associated (1685 nm), and lignin (2132 and 2267 nm), bands assigned to water (1927 nm) and non-lignin-related components lack direct relevance to the HTC process. In other words, the new variables created via dimensional reduction by PLS sufficiently preserved the variance of the original data and simultaneously revealed the unique features of each observation. For data with a high correlation among input variables, i.e., high multicollinearity, it has been reported that PLSR provided more stable results than OLSR^26^.

The PLSR model combined with NIRS is a promising approach for predicting the carbonization characteristics of lignin-derived hydrochars produced via HTC, which allows for non-destructive analysis.

## Conclusions

As the reaction severity in HTC increased, more carbon-intensive hydrochars were produced. NIRS is an effective tool for capturing information regarding the reaction severity, and PCA provided insights into some of the spectral regions that contained the information thereof. The PLSR models established with NIR data accurately predicted the carbonization characteristics of hydrochars, and the selection of the spectral region and spectral pretreatment by the second derivative improved the performance of the models. The results of this study demonstrated that the PLSR model combined with NIRS is a promising approach for the rapid and non-destructive prediction of the carbonization characteristics of kraft-lignin-derived hydrochars produced via HTC. The operational simplicity and speed of NIRS allow for real-time measurements; thus, this method can be applied to industry as an on-line measurement system in the HTC process or an at-line system if additional sample conditioning is required.

## Materials and methods

### Sample preparation

Hydrochars were produced by the HTC of kraft lignin, a byproduct of industrial-scale pulping for producing bleached hardwood pulp. In cooking and bleaching, a strong alkaline white liquor comprising sodium hydroxide, sodium sulfide, and chlorine dioxide is used. Suspensions of 5.6 g of lignin powder and distilled water (140 mL of distilled water, i.e., a solid-to-liquid ratio of 2/50), were hydrothermally carbonized.

### Hydrothermal carbonization

To produce hydrochars from lignin and to investigate the effects of temperature and reaction time on the carbonization characteristics of lignin in HTC, the suspension was placed in a glass liner and heated for 1, 2, 3, and 5 h in a heating mantle set at temperatures of 175 °C, 200 °C, 225 °C, and 250 °C, respectively. The heating rate was 2.6 °C/min, and the reaction time refers to the period in which the temperature is maintained constant after reaching each set temperature. At the end of the target reaction time, heating was stopped, and the reaction vessel was stored at room temperature for natural cooling. Subsequently, hydrochars, i.e., solid residues formed via HTC, were vacuum filtered and dried in an oven at 60 °C for 48 h. The dried samples were pulverized and then humidified at 25 °C and 60% relative humidity for elemental analysis and NIRS measurements.

### Elemental analysis

The carbonization characteristics of the hydrochars were investigated by quantifying C, H, N, and S using an elemental analyzer (Flash 2000, Thermo Fisher Scientific, Waltham, MA, USA). The C, H, N, and S in the sample reacted with pure oxygen at a high temperature of 850–950 °C and converted into CO_2_, H_2_O, N_2_, and SO_2_. These gases were purified, passed through a gas chromatography column, separated, and then quantified using a thermal conductivity detector.

### NIRS

NIR spectra were obtained from the HTC lignin and control samples using an NIR spectrometer (NIR Quest, Ocean Insight, Orlando, FL, USA) equipped with a tungsten-halogen light source and a reflection probe (QR400-7-VIS-BX, Ocean Insight, Orlando, FL) with an outer diameter of 6.35 mm and a core diameter of 0.4 mm. The spectra were collected at a wavelength of 870–2500 nm and an optical resolution of 6.6 nm; subsequently, 16 scans were averaged per scan. Three spectra were obtained per sample; hence, 51 spectra were obtained from the prepared samples. Prior to performing the multivariate analysis, the original NIR spectrum was normalized and second derivatized via Savitzky–Golay filter smoothing^27^ to 11 points using the fifth-order function. As regions under 1250 nm and beyond 2300 nm were noisy, the spectral range of 1250–2300 nm was used for the multivariate analysis.

### Multivariate analysis

PCA was performed to analyze the chemical changes in the lignin samples caused by HTC. PCA transformed the NIR data with a spectral range of 1250–2300 nm into a new orthogonal coordinate system comprising six components. The carbonization characteristics of the lignin and the spectral regions affecting it were investigated by analyzing the scores and loadings of the PCs.

PLSR was employed to establish models for predicting the carbonization characteristics of the lignin via HTC. Models that output the carbon content (C wt%), O/C ratio, and H/C ratio were designed using NIR spectral data as input variables. The optimal number of latent variables for the PLSR models was obtained using the leave-one-out cross-validation technique^28^. The performance of the PLSR models was evaluated using *R*^2^ and the RMSE for the calibration and prediction sets.

$$R^{2} = 1 - \left( {\sum\limits_{i} {(y_{i} - \hat{y}_{i} )^{2} } /\sum\limits_{i} {(y_{i} - \mu )^{2} } } \right),$$

$${\text{RMSE}} = \sqrt {\frac{1}{n}\sum\limits_{{i = 1}}^{n} {(\hat{y}_{i} - y_{i} )^{2} } } ,$$

where ${y}_{i}$ and ${\widehat{y}}_{i}$ are the measured and predicted values of the *i*-th observation, respectively, *μ* is the overall mean, and *n* is the number of observations in the calibration and prediction sets. The PCA and PLSR models were implemented using Python 3.8, with programming libraries for multivariate analysis.

## Data Availability

The datasets generated and/or analyzed during the current study are available from the corresponding author upon reasonable request.
